# Ethnoveterinary Study of Traditional Medicinal Plants Used for Livestock Healthcare in Kaffa Zone, Southwestern Ethiopia

**DOI:** 10.1155/tswj/7853406

**Published:** 2026-11-15

**Authors:** Tamirat Wato, Nigus Kalayou, Abera Haile, Salasib Atumo, Alemu Andualem, Ginjo Gitima, Mathewos Agize

**Affiliations:** ^1^ Department of Plant Science Bonga University Bonga Ethiopia bongau.edu.et; ^2^ Department of Biology Bonga University Bonga Ethiopia bongau.edu.et; ^3^ Department of Chemistry Bonga University Bonga Ethiopia bongau.edu.et; ^4^ Department of Geography and Environmental Studies Bonga University Bonga Ethiopia bongau.edu.et; ^5^ Department of Biology Wolaita Sodo University Wolaita Sodo Ethiopia wsu.edu.et

**Keywords:** medicinal plants, native people, plant parts, traditional knowledge

## Abstract

Traditional medicinal plants are widely used in Ethiopia, yet their application in the Kaffa zone remains undocumented. This study is aimed at assessing and identifying medicinal plants used for treating livestock ailments to help preserve indigenous knowledge and filling the information gap. The traditional medicinal plants for the treatment of livestock disease data were collected between December 2024 and March 2025 across four districts using structured interviews, semistructured interviews, focus group discussions, and questionnaires. Specimens were verified at the National Herbarium using taxonomic keys; we used Microsoft Excel for data analysis. Informant consensus factor, preference ranking, direct matrix ranking, and fidelity level were used for quantitative ethnobotanical data techniques. The results of this study showed that the Solanaceae family (21.7%) was the highest number of plant families, followed by Euphorbaceae and Fabaceae (17.4% and 8.7%, respectively). The results of the growth habits of medicinal plant species showed that herbs (36.36%) were the most common habit form, followed by shrubs and trees (27.77% and 27.28%, respectively). The result of plant parts used in the study area revealed that the leaf (37.95%) was the most frequently used plant part for the preparation of the remedy. The main methods of plant parts remedy preparation were sapping (14.705%), followed by crushing (11.675%), and crushing and mixing (11.675%). The result of diseases treated in the study area showed that *Nicotiana tabacum* L. and *Ehretia cymosa* Thonn. were commonly used to treat the highest number of livestock ailments, such as lack of milk production, plant toxins, and leech infection, respectively. This study is generally in line with previous research on plant family distribution, confirming the prominence of Solanaceae, Euphorbiaceae, and Fabaceae in disturbed or semidisturbed environments. The predominance of leaves in this study is consistent with existing literature, confirming their widespread utilization across various domains, particularly in traditional medicine and food. The plant habit forms observed in this study reflect ecological processes such as succession and disturbance dynamics. Herbs are more common in disturbed or early‐stage ecosystems, whereas shrubs and trees dominate mature, undisturbed areas. The study′s identified hierarchy of remedy application oral (51.28%), dermal (30.77%), and nasal and spray (5.14%) aligns with global drug delivery trends. This study′s findings on plant toxins, leech infections, scabies, and insect repellent use are consistent with prior research, reinforcing the importance of these health concerns in specific populations. Therefore, researchers should prioritize documentation of traditional knowledge in detail, through participatory methods that engage local healers and livestock owners.

## 1. Introduction

In Africa, the majority of the population receives some of their basic health care from traditional medicine [1–3]. Actually, Ethiopian flora has led to studies assigning it as a world center of variety and the country [4, 5]. Ethiopia has a large number of ethnicities and an exceptionally diverse flora, including numerous local or restricted plant species employed in various traditional medical procedures [3, 6]. The majority of the evidence is still in the palms of traditional and knowledge healers, either lost or passed down orally, and thus ethnobotanical research attempts to attain and document the knowledge of the healers in the community to preserve it for future use [7, 8]. Ethiopia has a long history of employing traditional medicinal plants to treat various maladies, as documented in the country′s medicinal religious documents [3, 9].

Ethnobotany is described as the study of how individuals with a specific culture or religion employ therapeutic herbs. Since the outset, the local community has improved its own clearly defined knowledge of plant utilization, protection, and monitoring [10, 11]. The World Health Organization recognized the value of traditional medicine as a primary source of treatment for the first time in 1978. According to WHO [12], traditional medicine encompasses all knowledge and practices, whether explainable or not. According to [13], traditional medicine is practiced all over the world based on locally effective natural resources and traditional knowledge, and plant products to heal ailments have been explored for over 5000 years.

Traditional medicinal plants play a significant role in ethnoveterinary medicine by supporting livestock health and improving animal productivity [14]. They are widely used by rural communities to treat and prevent various livestock diseases, especially in areas where access to modern veterinary services is limited. Ethnoveterinary medicinal plants are locally available, affordable, and culturally accepted by livestock owners [14]. They are also used as natural remedies against parasites, insect pests, wounds, alimentary tract and metabolic disorders, and infectious diseases. In addition, the use of traditional medicinal plants helps preserve indigenous knowledge, reduces dependence on expensive synthetic drugs, and contributes to sustainable and environmentally friendly livestock management practices.

The study of ethnobotany is important because it allows for direct contact with the veracity of information on the use of plants, both wild and cultivated, for agricultural implements, fodder, medicine, food, fiber, and profits around the world [8, 15]. Ethiopia′s diverse cultural traditions and varied natural feature arrangement make it a biodiversity asset. In order to successfully describe, identify, scale, and improve traditional medicine plants, ethnobotanical research is essential in bioresource areas [16]. In Ethiopia, medicinal plants were crucial for treating a variety of illnesses [17]. However, research shows that a sizable population in various regions of Ethiopia uses traditional medicinal plants.

The Kaffa zone study provides new, localized ethnoveterinary data by documenting unique medicinal plants and plant ailment uses not previously recorded in other Ethiopian regions. It also offers quantitative insights such as informant consensus factor (ICF), fidelity level (FL), direct matrix ranking (DMR), preference ranking, and conservation‐relevant information specific to livestock remedies. Additionally, it highlights culturally distinct knowledge transmission patterns and compares Kaffa practices with other districts, revealing the zone′s unique ethnoveterinary profile. However, there is limited information regarding the use of traditional medicinal plants for ethnoveterinary in the study area in the literature. Thus, this study is highly relevant because livestock production is a major component of rural livelihoods and household income in the study area. Most communities depend on livestock for food, draft power, manure, and economic security. In the study areas, access to modern veterinary services is limited due to inadequate infrastructure, long travel distances, and high treatment costs. As a result, local communities have long relied on ethnoveterinary knowledge and medicinal plants to manage livestock diseases. Therefore, documenting and evaluating ethnoveterinary practices is important for preserving indigenous knowledge and supporting sustainable livestock health management. That is why ethnobotanical knowledge of the nation′s medicinal plants and documentation of cultural inheritance in its entirety are ways to preserve indigenous knowledge of medicinal plants before it is lost and irretrievably lost. For that reason, the present study is aimed at filling this information and knowledge gap in the study area concerning the assessment and identification of traditional medicinal plants to treat the health problems of livestock in the study area.

## 2. Materials and Methods

### 2.1. Location, Description, and SocioCultural of Kaffa Zone

The Kaffa zone is located 456 km southwest of Addis Ababa, Ethiopia. This zone is renowned for its rich biodiversity, cultural heritage, and historical significance. It is particularly well‐known for being the origin of coffee (*Coffea arabica*), one of the world′s most popular beverages. The following is a detailed description of the Kaffa zone study area based on geographical, environmental, cultural, and socioeconomic aspects. The people of Kaffa have extensive knowledge of medicinal plants and traditional health practices. Plants are used for treating a wide range of ailments, including alimentary tract and metabolic illnesses, respiratory problems, and infections. Some plants in the Kaffa zone are considered sacred, and certain forests are reserved for ritual and spiritual practices. These sacred areas are protected by the local community and play a central role in local cultural and spiritual life. Traditional healers in Kaffa are recognized for their expertise in using plants to treat various illnesses. These healers often possess deep knowledge passed down through generations, contributing significantly to the region′s ethnobotanical knowledge.

#### 2.1.1. Geographical Location and Administrative Boundaries

Kaffa Zone is situated in the southwestern part of Ethiopia, predominantly within the SNNPR. It is bordered by South Central Ethiopia to the south, Sheka Zone to the west, Bensa (Sidama Zone) to the north, and Bench Maji Zone to the east. It is located approximately between 6° 00 ^′^ and 7° 30 ^′^ N latitude and 35° 00 ^′^ and 37° 00 ^′^ E longitude. The capital of Kaffa Zone is Bonga, which is located in the central part of the zone (Figure 1).

**Figure 1:**
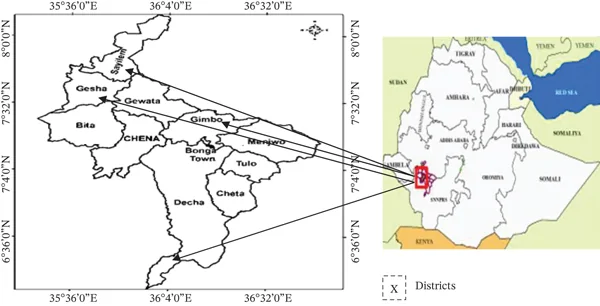
Map of the study areas. Data source: CSA [18] Population and Housing Census Map.

#### 2.1.2. Topography and Elevation

The zone is characterized by diverse topography, ranging from lowland areas to highlands. The altitude varies widely from 500 m in the lowlands to 2400 m in the highlands. The area features numerous mountains, plateaus, and valleys. The Bonga Mountains are a prominent feature, influencing local climate and vegetation. The zone is home to several rivers, including the Gibe River, which plays a critical role in irrigation and water supply.

#### 2.1.3. Climate and Ecosystems

Kaffa zone enjoys a tropical highland climate, which is conducive to the growth of various crops, including coffee. The climate is influenced by both altitude and the presence of tropical forests. The region experiences moderate rainfall, typically between 1500 and 2000 mm annually. The zone is well‐known for its dense tropical rainforests and montane forests, which are home to diverse plant species, many of which are used in traditional medicine. The forests also include wild relatives of the coffee plant, which have ecological and economic importance. The Kaffa zone is home to the Kaffa Biosphere Reserve, a UNESCO‐designated biosphere reserve that includes large tracts of rainforests and endemic species of plants and animals. Kaffa′s forests contain a variety of medicinal plants, edible plants, and wild species, including endemic trees, shrubs, and herbs. The region is also rich in wildlife, such as monkeys, antelopes, and various bird species, including the Ethiopian bush‐crow.

#### 2.1.4. Cultural and Ethnic Composition

The zone is ethnically diverse, with several indigenous groups residing in the area. The major ethnic groups include the Kaffa, Sheka, Bench, Maji, and Gamo peoples. The predominant language spoken in the Kaffa zone is Kaffa (also known as Kaffa or *Kafficho*). However, Amharic and Sheko are also spoken by some groups. Traditional knowledge related to the use of medicinal plants, herbal remedies, and cultural practices is passed down through generations. Elders and local healers (often referred to as “Wogebas”) hold a significant role in the community by providing healthcare through traditional methods and local plant resources.

#### 2.1.5. Agriculture and Economy

The economy of the Kaffa zone is primarily agricultural. The region is especially famous for its coffee, particularly *Arabica coffee*, which is native to the region. Coffee is grown in both smallholder farms and larger commercial plantations. Kaffa is considered the “cradle of coffee.” The wild coffee species, *C*. *arabica*, is believed to have originated in the forests of Kaffa, and the region continues to be a major producer of high‐quality coffee. In addition to coffee, the region produces a variety of crops such as teff, maize, sorghum, barley, wheat, beans, and various tuber and root crops. Livestock farming is also practiced, with cattle, goats, and sheep being raised in the zone. On average, there are 8–11 cattle, 1–5 sheep, 1–4 goats, and 3–6 poultry chickens per livestock owner in the study areas. And also, there are many companion animals in the region, such as cats and dogs.

#### 2.1.6. Biodiversity and Conservation

The Kaffa zone is home to several important ecosystems, including tropical forests, montane ecosystems, and wetlands. These ecosystems harbor a wide variety of plant and animal species, some of which are endemic to the region. The Kaffa Biosphere Reserve is recognized for its biodiversity conservation efforts. It protects key species and habitats, contributing to the preservation of Ethiopia′s natural heritage. Efforts to conserve indigenous plant species, especially medicinal plants, have been crucial in maintaining the ecological balance and cultural traditions of the local communities.

### 2.2. Preliminary Survey and Study Sites Selection

The initial survey was conducted from April to September 2024. The objective of this preliminary survey was to gain a comprehensive understanding of the region′s agroecological characteristics, assess the current state of vegetation, collect insights into the indigenous knowledge of local communities regarding the various uses of plants, and evaluate the accessibility of medicinal plants for the treatment of livestock diseases. The actual data collection was conducted between December 2024 and March 2025. In this study, the purposive sampling method was utilized to select the administrative level (Kebeles) from the four districts. The districts were selected rich diversity of medicinal plants, presence of traditional healers and indigenous knowledge, availability of ethnobotanical information, cultural diversity and traditional practices, accessibility and security, agroecological variation, and recommendations from local authorities. Priority was given to the accessibility of medicinal plants for the treatment of livestock diseases, and a documented history of medicinal plants for the treatment of livestock diseases. The selection process was further guided by previous data gathered by Gitima et al. [19] from local healthcare practitioners, participants in focus group ethnobotany research and applications for discussions, and traditional healers. Consequently, three subdistricts were selected from each district for the study (Table 1).

**Table 1:** The lists of districts and selected administrative levels with their household numbers.

S. No.	Districts	Coordinates	Administrative levels (kebeles)	No. of households
1.	Gimbo	7°20 ^′^N 36°10 ^′^E	Cheraba	3575
			Tephibuti	2318
			Kuti	4019

2.	Sayilem	7°54 ^′^50 ^″^N 35°51 ^′^12 ^″^E	Tareko	4290
			Shunga Shunit	3679
			Agaro	2586

3.	Goba	7.000°N36.000°E	Shashi	2346
			Angela	2195
			Diishi	2753

4.	Gesha	7°40 ^′^N 35°50 ^′^E	Ebeta	3105
			Daniti	2765
			Mashami	3284

Total	4		12	36,915

### 2.3. Data Collection Tools and Methods on Ethnobotanical Study

In ethnobotanical studies, data collection is crucial for gathering information about how local or indigenous communities use medicinal plants for the treatment of livestock diseases in the study areas. The ethnoveterinary methodology employed in this study was developed based on established ethnobotanical and ethnoveterinary research approaches described in the literature. Data were collected using widely accepted methods, including structured and semistructured interviews, focus group discussions (FGDs), and questionnaires, following standard procedures commonly used in ethnoveterinary research to ensure the reliability and reproducibility of the findings. This study used the following tools and methods to gather the data:

#### 2.3.1. Structured Interviews

These are guided, formal interviews where researchers ask a predefined set of questions to participants. Structured interviews are useful for gathering quantitative data about plant uses, such as which plants are most commonly used, how frequently they are used, and for what ailments. They are often used to assess knowledge of medicinal plants, plant parts used, preparation methods, and frequency of use. Interview guides include questions related to plant uses, preparation methods, and cultural practices.

#### 2.3.2. Semistructured Interviews

These interviews are less rigid than structured interviews and allow for more open‐ended responses. Researchers can explore topics more deeply and adjust the questions based on the participant′s responses. This method allows the researcher to explore both the depth of knowledge and personal and community perspectives about plants, uses, and cultural meanings. Flexible interview guides with key topics, but leaving space for exploration of emergent themes.

#### 2.3.3. FGDs

FGDs are group interviews where a researcher guides a conversation about plant use among a group of community members. This method encourages discussion and can help gather diverse views on plant knowledge and use. It is used to identify indigenous knowledge about medicinal plants and to understand how plant knowledge is shared and transmitted. Thus, a facilitator has contributed to guidance through audio recorders, photo capturing, and note‐taking tools.

#### 2.3.4. Questionnaires

Questionnaires are structured forms with a series of questions, often combining both closed‐ended and open‐ended questions. They can be used to gather data on plant species, their uses, and community knowledge on a large scale. They are typically used for large‐scale surveys where multiple people from a community are interviewed systematically.

### 2.4. Plant Voucher Specimens Collection, Identification, and Herbarium Preparation

During field excursions, medicinal plant specimens were collected with herbalists. Three specimens of each species were gathered, labeled with a collection number and collector names. They were arranged between blotting paper sheets, secured with a specimen presser, and dried in sunlight while checking for insect infestations. A guidebook on Ethiopia′s useful trees and shrubs assisted in identification [20]. Specimens were verified at the National Herbarium (ETH) using taxonomic keys. Further, identification methods were cross‐referenced with various databases for African applications, PlantNet identification, experts, recognitions, and comparison with authenticated specimens, Plants of the World Online, the Royal Botanic Gardens Kew, and the World Checklist of Selected Plant Families. For complete Latin binomial nomenclature, the JSTOR Global Plants website was consulted. The specimens were deposited at the National Herbarium (ETH) of Ethiopia for future research.

### 2.5. Informant Selection

The research involved conducting interviews with 400 participants, ranging in age from 18 to 85 years. Nine individuals were selected from each of the 12 administrative levels (kebeles). Consistent with prior studies [10, 21], 50 general informants (nonexpert healers) were chosen using simple random sampling from the indigenous peoples in the area. Additionally, about 24 key informants (expert healers) were selected through purposive sampling based on specific criteria. Participants were grouped into three distinct age categories: young adults (18–35), middle‐aged individuals (36–55), and seniors (56–85). The main focus of the study was to explore the intergenerational transfer of indigenous medicinal plants knowledge, with a particular emphasis on individuals under the age of 30 [8, 22].

### 2.6. Data Analysis

Frequency analysis was conducted using Excel Software (tables, bar graphs, and pie charts) to analyze the ethnobotanical data of the medicinal plants for the treatment of livestock ailments, as well as various related healing experiences.

### 2.7. Quantitative Ethnobotanical Data Techniques

Quantitative ethnobotanical data techniques such as the ICF, preference ranking, DMR, and FL were utilized for data analysis. These ethnobotanical indices were complemented with statistical tests, models, and ordination methods to illustrate the relationships between plants and humans [23]. The data were then further processed and analyzed using appropriate statistical tools in SAS to uncover patterns and structures in human‐plant interactions. The use of numerical methods in ethnobotanical research was found to be an effective approach for representing the structure of plant–human relationships [24–26].

#### 2.7.1. ICFs

The reliability of the recorded information was verified by contacting informants at least twice regarding the same concepts, allowing for an assessment of the data′s dependability collected during interviews. Any original information was deemed unreliable if it contradicted the consensus of the informants. To evaluate the level of agreement among informants about the reported treatments for specific illnesses, the ICF was calculated for each category [27]. This calculation was performed as follows:

ICF=Nur−NtNur−t,

where Nur is the number of informant use reports for a specific plant‐use category, and Nt is the total number of taxa or species used for that plant‐use category across all informants. The index has a range of 0–1, where values close to 1 indicate that informants strongly agree that the same species is used [21].

#### 2.7.2. Preference Ranking

In this study area, the eight key informants assessed the effectiveness of the various medicinal plants. The plants were ranked according to the informants′ evaluations, with scores ranging from 1 (least effective) to 7 (most effective). The overall ranking for each plant was determined by summing the scores assigned to each species [10, 28].

#### 2.7.3. DMR

To assess the various uses of medicinal plants, eight key informants ranked seven species across eight categories, such as food, firewood, and medicinal uses. The ranking scale ranged from 0 (not used) to 5 (most beneficial use) [28]. The total utility ranking for each species was calculated by summing the average scores assigned to them.

#### 2.7.4. FL

FL, as proposed by [29, 30], was employed to assess the relative therapeutic potential of medicinal plants in addressing livestock and human health issues. The calculation of the FL was conducted using the following formula:

FL %=IPIU∗100.

Hence, FL is a metric used to assess the healing potential of medicinal plants for specific health conditions. It is calculated by the number of informants who independently cite a plant for treating an ailment (IP) divided by the total number of informants reporting the plant for that disease (IU). A high FL indicates that the plant is highly regarded for its effectiveness and may contain bioactive compounds with therapeutic properties, making it a priority for further pharmacological research and drug development.

### 2.8. Ethical Considerations

Before the field excursions, the Department of Biology and Plant Science sent formal correspondence to key stakeholders, including the district agriculture office, district administrators, and kebele leaders, to gain their support for the study. Throughout the research process, we placed a strong emphasis on upholding ethical standards. We made sure to inform all herbalists involved that the study was being conducted for academic purposes. Ethical approval was obtained to guarantee confidentiality before conducting interviews. Additionally, we were committed to safeguarding the local communities′ knowledge, respecting their secrets and cultural taboos while documenting our findings.

## 3. Results and Discussion

### 3.1. Sociodemographic Characteristics

The analysis indicated that there were statistically significant differences (*t* = 14.7, *p* ≤ 0.05) among the genders of the respondents (Table 2). Male informants reported a higher average knowledge score (*M* = 11.84, SD = 6.08) than female informants (*M* = 4.94, SD = 6.2) (Table 2).

**Table 2:** Sociodemographic characteristics of the respondents.

Variable	Groups	Number of respondents	Mean	SD	*t*‐values	*p*
Gender	Male	288	11.84	6.08	14.7	*p* ≤ 0.05
	Female	120	4.94	2.54	6.2	

Educational status	Illiterate	342	14.06	7.22	14.5	*p* ≤ 0.05
	Read/write only	31	1.27	0.66	1.6	
	Formal education	19	0.78	0.41	0.9	
	Tertiary education	8	0.33	0.17	0.4	

Age group	18–30 years	43	1.77	0.91	2.2	*p* ≤ 0.05
	31–45 years	262	10.78	5.53	13.4	
	46–65 years	75	3.08	1.58	3.8	
	66–85 years	20	0.83	0.43	1.1	

Ethnic group	Chara	30	1.24	0.64	1.5	*p* ≤ 0.05
	Kaffa	321	13.2	6.78	16.4	
	Na′yee	19	0.78	0.41	0.9	
	Amhara	16	0.66	0.34	0.8	
	Oromo	14	0.58	0.29	0.7	

The substantial gap in the mean scores suggests that male participants possess more knowledge or awareness regarding the subject matter assessed in the study. This finding implies that gender may be an influential factor affecting knowledge acquisition or access to information within the study population. The results highlight the need for further investigation into the underlying causes of this disparity, such as differences in educational opportunities, cultural roles, or access to resources, and call for strategies to bridge the gender gap in knowledge and information sharing.

The analysis revealed a statistically significant difference in knowledge scores among respondents with varying educational backgrounds (*p* ≤ 0.05), indicating that the level of education had a measurable impact on the knowledge reported by the participants (Table 2). Remarkably, the results showed that informants with no formal education (illiterate participants) had the highest average knowledge score (*M* = 14.06, SD = 7.22), whereas those with tertiary‐level education had the lowest average score (*M* = 0.33, SD = 0.17) (Table 2). This counterintuitive finding, supported by a high *t*‐value of 14.5, suggests that knowledge related to the subject under investigation (likely traditional or indigenous knowledge) may not be strongly associated with formal education (Table 2). In fact, it appears that individuals with less or no formal education possess significantly more practical or experiential knowledge in this area.

This pattern could be attributed to the nature of the knowledge being assessed, possibly local, cultural, or traditional practices which are often passed down orally and informally through generations, rather than through structured educational systems. Those with higher levels of formal education might rely more on scientific or modern knowledge systems, which may not emphasize or value traditional knowledge in the same way. These findings emphasize the importance of recognizing and integrating indigenous knowledge systems into formal research and development efforts, especially in areas where traditional practices play a critical role in community life, such as agriculture, livestock care, or natural resource management. In line with the present finding, Gitima et al. [19] also reported the same insights. It also calls for inclusive approaches that respect and incorporate the contributions of individuals from all educational backgrounds.

The analysis showed statistically significant differences in knowledge scores across different age groups of respondents (*p* ≤ 0.05), indicating that age had a meaningful impact on the level of knowledge reported (Table 2). Specifically, the age group between 31 and 45 years demonstrated the highest average knowledge score (*M* = 10.78, SD = 5.53), with a corresponding *t*‐value of 13.4 (Table 2). In contrast, the oldest age group (66–85 years) recorded the lowest average knowledge score (*M* = 0.83, SD = 0.43), with a *t*‐value of 1.1 (Table 2). These results suggest that individuals in their middle age (31–45 years) possess greater knowledge regarding the subject under investigation, whereas knowledge levels tend to decline significantly among the elderly.

The relatively high knowledge scores among the 31–45 age group may be attributed to their active engagement in practical, community‐based activities, making them more involved and experienced in the topic area, possibly related to agriculture, traditional medicine, or other local practices. This age group may also represent a balance between physical ability, cognitive performance, and accumulated life experience, making them key knowledge holders in their communities. In conformity with this study, Gitima et al. [19] explained in a similar way. On the other hand, the low scores among the oldest participants could reflect age‐related factors such as cognitive decline, limited recent practice or involvement, or reduced memory retention. It may also suggest that although older individuals might have once held valuable knowledge, much of it may not have been retained, practiced, or passed on effectively.

These findings underscore the importance of targeting the middle‐aged population in knowledge preservation and dissemination strategies, while also creating opportunities for intergenerational knowledge transfer to ensure that traditional wisdom is not lost over time.

The analysis revealed statistically significant differences (*p* ≤ 0.05) in knowledge scores among the different ethnic groups of the respondents, indicating that ethnicity had a meaningful influence on the level of knowledge reported (Table 2). Among the ethnic groups assessed, informants from the Kaffa ethnic group demonstrated the highest average knowledge score (*M* = 13.2, SD = 6.78), with a strong statistical significance (*t* = 16.4). In contrast, respondents from the Oromo ethnic group recorded the lowest average knowledge score (*M* = 0.58, SD = 0.29), with a relatively weak *t*‐value of 0.7 (Table 2). This suggests that individuals from the Kaffa ethnic group possess considerably more knowledge related to the subject under study, likely traditional practices or ethnobotanical knowledge, compared with other ethnic groups, particularly the Oromo. This disparity may be attributed to cultural differences, varying levels of dependence on traditional knowledge systems, or differences in exposure to or involvement in the practices being evaluated.

The high knowledge scores among the Kaffa may reflect a deeper cultural connection to indigenous practices and long‐standing familiarity with traditional systems passed down through generations. On the other hand, the relatively low scores among the Oromo group could imply less engagement with or emphasis on such knowledge in their daily lives or community structures. These findings highlight the critical role that cultural background and ethnic heritage play in shaping traditional knowledge systems. They also emphasize the need to consider ethnic diversity when designing knowledge preservation and dissemination programs, ensuring that valuable insights from knowledge‐rich communities like the Kaffa are recognized and preserved.

### 3.2. Relevant Traditional Medicinal Plants Used to Treat Animal Ailments

The relevant medicinal plants used to treat animal ailments are illustrated in Table 3.

**Table 3:** List of medicinal plants used to treat animal diseases.

Scientific name	Family	Local name	Habit	Habitat form	Parts used	Disease treated	ATCvet group	Types of animals treated	Mode of application and administration	Application route
*Cymbopogon citratus* (DC.) Stapf	Poaceae	Teji sar (A)	H	Plantations, tropical, and subtropical areas	Leaf	Snake bite	QI	Cattle	The leaves are prepared and drunk as tea.	Oral
								Goat		
								Sheep		
		Tojoo (K)				Alimentary tract and metabolic, high blood pressure, and common cold	QA, QC, QR	Human and livestock	The leaves are prepared and drunk as tea.	Oral
*Croton macrostachyus* Hochst. ex Delile.	Euphorbiaceae	Bisana (A)	T	Evergreen forests, bushlands, and woodedlands	Leaf	Scabies	QP	Cattle	The leaf of *C*. *macrostachyus* is crushed and applied to the affected skin for three to 4 consecutive days.	Dermal
								Goat		
		Waagoo (K)						Sheep		
					Twig	Dislocation of a body part	QM	Cattles	The dislocated part is tied with twigs until healed.	Dermal
								Sheep		
*Erhetia cymosa* Thonn.	Boraginaceae	Game (A)	T	Evergreen forests, bushlands, and wooded savannah	Twigs and leaf	Increases milk production	QH	Cattle	The entire leaves are chopped and crushed, then mixed with some salt and water before being given to the animals.	Oral
								Sheep		
		Wagaammo (K)						Goats		
*Phytolacca dodecandra* L′Herit.	Phytolaccaceae	Endod (A)	S	Forests, bushland, grasslands, and t hickets	Leaf	Rabies	QI	Cattle	The leaves are crushed together with the leaves of *C*. *mycrostachyus*, then squeezed to extract the juice. One cup of the juice is mixed with “tella” and administered to the animal.	Oral
								Sheep		
		Yingamoo (K)						Goats		
						Scabies and external parasite infestation	QP	Cattle	The leaves are crushed with a small amount of water to make a paste, which is applied to the skin. After 30 minutes, the skin is rinsed off.	Dermal
								Sheep		
								Goats		
*Melicoccus bijugatus* Jacq.	Sapindaceae		S	Arid and semiarid	Leaf	Sore on cattle	QD	Cattles	The leaves are dried on a hot iron plate, ground into powder, and then applied to the affected area.	Dermal
								Sheep		
*Rhamnus prinoides* L′Herit.	Rhamnaceae	Gesho (A)	S	Forests, mountains, and plantations	Leaf	Plant toxin (toxicosis)	QA	Cattle	The leaves are crushed into a powder and mixed with barley malt, oil, or dissolved soap. One or two cups of the mixture are then given to the poisoned animal.	Oral
								Sheep		
		Geeshoo (K)						Goat		
					Twig with leaf	Increase milk production	QH	Cattles	The twigs and leaves are crushed and mashed, then salt is added before mixing with boiled water. After cooling, the mixture is given to the animals.	Oral
								Sheep		
								Goat		
*Nicotiana tabacum* L.	Solanaceae	Timbaho (A)	H	Forests, wetlands, and mountains	Leaf and root	Plant toxin	QA	Cattle	The leaves and roots are dried and powdered, then mixed with salted water. One cup of this solution is given to the poisoned animal for 1 day.	Oral
		Qumbafoo (K)						Sheep		
								Goat		
					Leaf	Leech infestation	QP	Cattle	The leaves are crushed and squeezed, and a cup of the extracted solution is applied nasally.	Nasal
						Leech infestation	QP	Cattle	The crushed and baked leaves are pounded, mixed with half a liter of water, and given to the affected animal.	Oral
						Trypanosomiasis	QP	Cattle	The leaves are crushed and baked, then mixed with a solution and administered to the sick animal.	Oral
*Ruta chalepensis* L.	Rutaceae	Tena′Adam (A)	H	Plantations, thickets, and mountains	Leaf	Coccsidiosis	QP	Poultry	The entire plant, root of *Justicia schimperiana*, and bark of *C*. *mycrostachyus* are pounded together into a paste, which is then mixed with “enjera” or water and given to the chicken.	Oral
		Chadiramoo (K)								
*Manihot esculenta* Crantz	Euphorbiaceae	Enchet‐boye (A)	T	Plantations and fence	Leaf	Cholera	QJ	Poultry	The leaves are chopped and combined with chalk powder, then added to water and given to the poultry.	Oral
		Occinoo (K)		Forests and wetlands						
*Solanum marginatum* L. f.	Solonaceae	Embuay (A)	S	Roadsides, forest, and wetlands	Fruit	Urinary retention	QG	Cattle	The fruits are crushed and pounded, then two to three spoonfuls of the powder are mixed with cold water. A cup of this solution is given to the sick animal.	Oral
								Sheep		
								Goat		
*Ricinus communis* L.	Euphobiaceae	Gullo (A)	T	Roadsides and forests	Root	Sudden sickness	QV	Cattle	The roots of *R*. *communis* and *J*. *schimperiana* are pounded and mixed with cold water, and a cup of the resulting solution is given to the sick animal.	Oral
		Xesso (K)								
					Fruit	Anthrax	QJ	Cattle	The dried fruits are ground into a powder, mixed with cold water, and a cup of the solution is given to the sick animal.	Oral
*Allium sativum* L.	Alliaceae	Nech‐Shinkurt (A)	H	Plantations	Bulb	Thelaziasis (eye disease)	QS	Cattle	The bulbs are crushed, squeezed, and filtered, then mixed with soot to form a paste that is applied to the affected area.	Optical
		Dooqishoo (K)								
						Foot and mouth disease	QI	Cattle	*A*. *sativum* L. is crushed and mixed with honey, and then the paste is applied to the affected area.	Dermal
						Newcastle disease	QI	Poultry	The bulb is crushed, combined with “enjera,” and given orally.	Oral
*Calpurnia aurea* (Alt.) Benth.	Fabaceae	Hetsawets (A)	T	Forests and bushlands	Seed	Salmonellosis	QJ	Cattle	A cup of seeds is ground into powder, mixed with salted cold water, and the solution is administered orally.	Oral
								Sheep		
								Goat		
					Leaf	*Escherichia coli* infection	QJ	Cattle	The leaves are crushed, squeezed, and filtered, and the extracted juice is administered orally.	Oral
								Sheep		
								Goat		
						Lichen simplex chronicus (skin disease)	QD	Cattle	The leaves are crushed and applied directly to the skin.	Dermal
*Cissus quadrangularis* L.	Vitaceae	Edible stemmed vine	C	Forests, thickets, grasslands, and termite mounds	Veldt grape	Obesity	QA	Human and livestock	*C*. *quadrangularis* is used to help prevent or reduce weight loss.	Oral
					Stem	Wound/ skin cut	QD	Cattle	The stem juice is applied to the injured area.	Dermal
*Dodonaea angustifolia* L. f.	Sapindaceae	Kitikita (A)	S	Arid and semiarid areas	Leaf	Sore on cattle	QD	Cattles	The leaves are dried on a hot iron plate, ground into a powder, and sprinkled onto the affected area.	Dermal
								Sheep		
					Twig	Dislocation of a body part	QM	Cattles	The dislocated part is tied with twigs until healed.	Dermal
								Sheep		
*Curcuma longa* L.	Zingiberaceae	Erd (A)	H	Forests and plantations	Rhizome powder	Insect repellent	QP	Insects	Sprinkling a solution of turmeric powder and water around all entry points of your house helps repel insects, ants, and termites.	Spray
		Turmeric								
					Whole	Hair fall, hair thinning, and other hair problems	QD	Human and livestock	The extracts of this plant are used as an ointment.	Dermal
*Euphorbia cactus* Ehrenb. ex Boiss.	Euphorbiaceae	Qulqual (A)	S	Desert and arid areas	Latex	Black leg	QI	Cattle	Latex is applied to the swollen area to prevent the spread of the disease. A small amount is also administered with “enjera.”	Dermaland oral
		Gaachoo (K)								
*Eucalyptus globulus* Labill.	Myrtaceae	Nech‐bahir zaf (A)	T	Forests, grasslands, and bushlands	Leaf	Avian cholera	QJ	Poultry	The leaf of *E*. *globulus* is ground into a powder, boiled in water, and the resulting solution is mixed with barley soup and fed to the chicken.	Oral
		Naacoo Barzafoo (K)								
*Lepidium sativum* L.	Brassicaceae	Fito (A)	H	Plantations and roadsides	Seed	Dysentery	QJ	Cattle	The seeds are crushed into a powder, mixed with finger millet bread, and given orally.	Oral
								Sheep		
						Diarrhea	QA	Cattle	The seeds of *L*. *sativum* are ground into a powder, mixed with crushed bulbs of *A*. *sativum*, and administered to the animal.	Dermal
						Bloating	QA	Cattle	Seeds of *L*. *sativum* and bulbs of *A*. *sativum* L. are crushed together, mixed with water, and given to cattle.	Dermal
*Clitoria ternatea* L.	Fabaceae	Butterfly pea	C	Grasslands and woodedlands	Leaves and dread root	Piles (*Kintarot*)	QA	Human and livestock	A decoction made from the entire plant is used to cleanse piles.	Dermal
					Leaf	Headache	QN	Sheep	Leaf juice is administered as nasal drops to relieve headaches.	Nasal
								Cattles		
*Bryophyllum pinnatum* (Lam.) Oken.	Crassulaceae	Bryophyllum	H	Forests, tropical, and subtropical areas	Leaves	Kidney stone	QG	Human and livestock	The rhizome of this plant can be used both to prevent and treat kidney stones.	Oral
*Datura stramonium* L.	Salanaceae	Astenagir (A)	H	Grasslands, roadsides, and plantations	Fruit	Hair loss	QD	Human and livestock	The fruit juice is applied to the scalp to treat dandruff and hair loss.	Dermal
		Nafinifo (K)								
					Whole parts	Insect repellent	QP	Insects	The growing plants help shield nearby plants from insects.	Dermal
*Solanum trilobatum* L.	Solanaceae	Embuay (A)	S	Along the roadside and plantations	Leaves	Cold, cough, and acute asthma	QR	Human and livestock	The young leaves and flowers are cooked and consumed as a salad.	Oral
					Flower					

*Note:* QP: antiparasitic products, insecticides, and repellents; QR: respiratory system; QC: cardiovascular system; QI: immunologicals; QA: alimentary tract and metabolism; QJ: anti‐infectives for systemic use; QD: dermatologicals; QS: sensory organs; QM: musculoskeletal system; QH: dystemic hormonal preparations (general hormonal products); QJ: anti‐infectives for systemic use; QG: genitourinary system and sex hormones; QV: various; QN: nervous system.

Abbreviations: A, Amharic; C, climber; H, herb; K, Kaffinoonoo; S, shrub; T, tree.

### 3.3. The Frequency of Plant Families

The results of this study showed that Solanaceae (21.7%) were the highest number of plant families, followed by Euphorbaceae (17.4%) and Fabaceae (8.7%) (Figure 2). The predominance of Solanaceae can be attributed to its widespread utilization in local medicine and its adaptability to diverse environmental conditions. This family includes a different of species known for their pharmacological properties, such as *Solanum* spp. and *Capsicum* spp., which have been extensively recorded for their therapeutic uses [31, 32].

**Figure 2:**
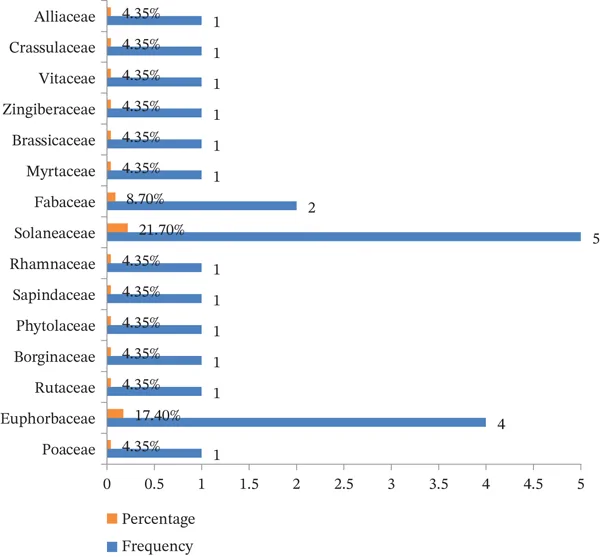
The frequency of plant families.

The prominence of Euphorbiaceae is consistent with other ethnobotanical studies conducted in tropical and subtropical regions, where species like *Euphorbia hirta* and *Jatropha curcas* are commonly used for treating different illnesses, including gastrointestinal disorders, skin infections, and inflammation [33, 34]. Euphorbiaceae species are also known for their latex content and secondary metabolites, which contribute to their medicinal significance.

Fabaceae was the third dominant in this study, still plays a significant role in traditional medicine systems. This family is well represented in global ethnobotanical records due to the abundance of nitrogen‐fixing species, nutritional value, and their inclusion in both medicinal and dietary uses [35, 36]. Plants like *Albizia lebbeck* and *Cassia fistula* are often cited in indigenous healing practices for their antimicrobial and anti‐inflammatory properties. The relative abundance of these families in ethnobotanical inventories reflects not only the richness of local flora but also the depth of indigenous knowledge regarding plant use. Variation in dominant families across studies may also result from differences in geographical, cultural, and ecological contexts [37, 38].

### 3.4. The Growth Habits of Medicinal Plant Species

The results of this study showed that herbs (36.36%) were the most common habit form, followed by shrubs (27.77%) and trees (27.27%) (Figure 3).

**Figure 3:**
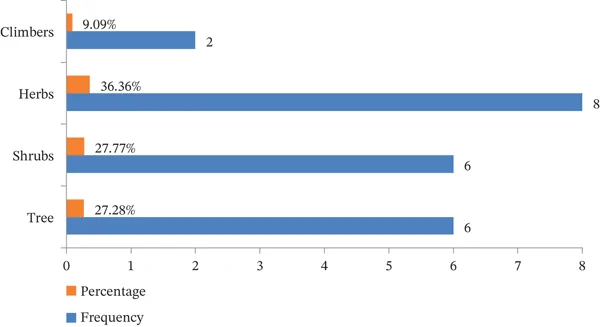
The growth habits of medicinal plant species.

The dominance of herbs in this study aligns with findings from several ecological studies (Figure 3). For instance, Smith [39] reported that herbaceous plants dominate disturbed or open ecosystems due to their faster growth rates and shorter lifespans, which allow them to establish quickly in areas with frequent disturbances. In a study by Jones [40] in tropical forest understories, a similar dominance of herbs was observed, particularly in areas with seasonal rainfall, where herbs can take advantage of the brief wet periods for rapid growth and reproduction. Shrubs were commonly found in ecosystems with moderate disturbances, such as fire‐prone areas or regions undergoing succession. Studies by Miller [41] have noted that shrubs tend to be resilient, able to regenerate quickly following disturbances and adapt well to a variety of soil conditions. This can explain the significant proportion of shrubs observed in this study.

Furthermore, Nguyen [42] found that shrubs often represent a transitional phase in forest succession, eventually giving way to trees as the canopy closes, which may also explain their substantial presence in the current study. Trees, although slightly less prevalent in this study, still represented a significant portion of the vegetation composition (Figure 3). Previous research, such as that by Gonzalez [43], has indicated that in mature, undisturbed ecosystems, trees tend to dominate due to their longevity and ability to form dense canopies that shade out other plants. In contrast, this study′s findings suggest that either disturbance or the particular environmental conditions may not yet favor tree growth as the most dominant form.

### 3.5. Habitat Forms

The result revealed that forest land emerged as the most frequently observed habitat Type 11 (21.15%); plantation areas followed closely with a frequency of 10 (19.23%); desert habitats had a frequency of 7 (13.46%); roadside and fence habitats recorded a frequency of 6 (11.53%), thickets had a frequency of 3 (5.8%); and mountains had a frequency of 2 (3.84%); finally, termite mounds had the lowest frequency (1, 1.92%). (Table 4).

**Table 4:** Habitat forms.

S. No.	Habitat forms	Frequency	Percentage
1.	Grassland	4	7.69
2.	Forestland	11	21.15
3.	Desert	7	13.46
4.	Plantations	10	19.23
5.	Bushlands	4	7.69
6.	Roadsides and fence	6	11.53
7.	Mountain	2	3.84
8.	Wetlands	4	7.69
9.	Tickets	3	5.8
10.	Termite mounds	1	1.92
	Total	52	100%

The dissemination of species across divergent habitat types is affected by a combination of environmental situations, ecological processes, and anthropogenic factors. The data show different frequencies and percentages of occurrence across 10 different habitat types, which provide insights into species preference and ecological richness. The result shows that forest land emerged as the most frequently observed habitat type, indicating a relatively high ecological richness and suitability for a wide range of flora and fauna (Table 4). Forest ecosystems support diverse species by providing shelter, food sources, and microclimatic stability [44, 45]. The higher frequency may also reflect the availability of structural complexity and layered vegetation that enhances niche diversity. Plantation areas followed closely with a high frequency. Although plantations are managed environments, they can still provide habitat opportunities, particularly when interspersed with natural vegetation or left seminatural at the margins [46, 47]. However, the biodiversity value of plantations typically depends on tree species diversity, age, and understory development. Desert habitats had a frequency indicating the adaptability of certain species to arid conditions (Table 4). Despite harsh environmental factors such as extreme temperature and limited water availability, desert ecosystems host specialized species adapted to these conditions [48, 49]. Roadside and fence habitats recorded the lowest frequency compared with the above habitat forms (Table 4). These linear habitats often serve as corridors for movement and dispersal, especially in fragmented landscapes [50–52]. They can inadvertently support biodiversity by connecting isolated patches, although they are also susceptible to pollution and human disturbance. Grasslands, bushlands, and wetlands each had a frequency of 4 (7.69%). Grasslands are critical for grazing species and ground‐nesting birds, yet their biodiversity can be threatened by overgrazing and agricultural conversion [53, 54]. Bushlands serve as transitional habitats, providing cover and foraging opportunities. Wetlands, despite their relatively low frequency, are globally important ecosystems for water filtration, flood control, and as breeding grounds for amphibians and birds [54, 55]. Thickets (frequency 3, 5.8%) are dense vegetation patches that offer protection and food for small mammals and birds. These habitats may support high species turnover due to their patchy nature. Mountains showed lower representation frequency (2, 3.84%), possibly due to inaccessibility or harsh climatic conditions at higher altitudes. Nonetheless, mountain ecosystems are vital for endemic species and often act as refugia during climatic shifts [56–58]. Finally, termite mounds had the lowest frequency (1, 1.92%). Although minimal in representation, termite mounds play a crucial role in ecosystem engineering by altering soil properties, nutrient cycling, and providing microhabitats for other organisms [59, 60].

### 3.6. Plant Parts Used

The result revealed that the Leaves were the greatest frequently used plant part for the preparations of remedy, which accounted for 11 (37.95%), followed by leaves and twig 2 (6.89%), bulb 2 (6.89%), fruit leaf 2 (6.89%), shoot 2 (6.89%), and whole parts 2 (6.89%) (Figure 4). This means showed that the leaves were the most frequent plant part identified, followed by fruits, bulbs, and whole plants (Figure 4), aligning with several previous studies in the field of plant morphology and ethnobotany. Leaves are often the primary plant part used in both traditional medicine and agriculture due to their abundance, accessibility, and roles in physiological processes such as photosynthesis, transpiration, and storage of essential nutrients. This frequent use of leaves may explain their prominence in various plant species studies. For example, a study by Fais and Era [61] found that leaves of many plant species are often the most utilized part, due to their wide range of bioactive compounds such as alkaloids, flavonoids, and phenolics, which contribute to their medicinal and therapeutic properties. Similarly, Balandrin [62] and Pant et al. [63] noted that leaves contain a variety of secondary metabolites, which may be why they are commonly selected in plant part analysis. Fruits, although not as frequently used as leaves, are also important for their nutritional and medicinal properties, as indicated in the work of Bussmann [64] and Rathnayake et al. [65], who reported that fruits are used extensively in food and traditional healing practices.

**Figure 4:**
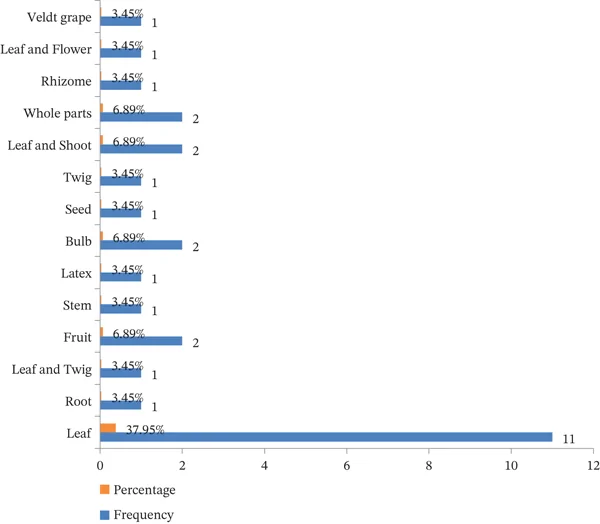
Plant parts used.

Bulbs, often associated with plant reproductive strategies, also have significant uses in food and medicine, particularly for their high‐energy content and ability to store nutrients (Figure 4). This is consistent with findings from Mann [66], who highlighted the role of bulbs in both culinary and therapeutic applications. Whole plants, being less frequently represented in this study, may reflect the complexity of harvesting and using all parts of a plant (Figure 4). However, whole plants are essential in certain contexts, such as in the preparation of herbal concoctions or when an integrated approach to plant usage is necessary for specific therapeutic effects [67].

### 3.7. Modes of Remedy Preparation

This result showed that the main methods of plant parts remedy preparation were sapping (14.705%), followed by crushing (11.675%), and crushing and mixing (11.675%) (Table 5). In this study, the most common modes of preparation observed were sapping, crushing, crushing and mixing, crushing and powdering, and mixing and powdering (Table 5). These results align with similar research conducted in the field of (insert field or specific study area here), where various preparation methods have been highlighted for their efficiency and effectiveness in a specific purpose.

**Table 5:** Modes of remedy preparation.

S. No.	Preparations methods	Frequency	Percentage
1.	Chopping	1	2.95
2.	Drying and powdering	1	2.95
3.	Crushing	4	11.675
4.	Crushing and powdering	3	8.825
5.	Chopping and mixing	1	2.95
6.	Mixing and powdering	3	8.825
7.	Crushing, squeezing, filtering, and mixing	1	2.95
8.	Crushing and mixing	4	11.675
9.	Grinding and powdering	2	5.883
10.	Crushing and rubbing	1	2.95
11.	Tying (bone setting)	1	2.95
12.	Ointing	2	5.883
13.	Decoction	1	2.95
14.	Sapping	5	14.705
15.	Scalping	1	2.95
16.	Sprinkling and powdering	1	2.95
17.	Sapping and mixing	1	2.95
18.	Cooking	1	2.95
	Total	34	100%

### 3.8. Diseases Treated for Livestock

The result showed that *Nicotiana tabacum* L. and *Erthetia cymosa* Thonn. were commonly used to treat the highest number of livestock ailments, such as lack of milk production, plant toxins, and leech infection, respectively, and *Datura stramonium* L. was commonly used to treat insect repellents (Table 6).

**Table 6:** Disease treated.

S. No.	Diseases	Frequency	Percentage
1.	Increase milk production	2	5.263
2.	Plant toxins	2	5.263
3.	Leech infection	2	5.263
4.	Trypanomiasis	1	2.63
5.	Coccsidiosis	1	2.63
6.	Throat infection	1	2.63
7.	Cholera	1	2.63
8.	Rabbies	1	2.63
9.	Scabies	2	5.263
10.	Sudden sickness	1	2.63
11.	Anthrax	1	2.63
12.	Thelaziasis	1	2.63
13.	Foot and mouth ailment	1	2.63
14.	Newcastle ailment	1	2.63
15.	*E*. *coli* infection	1	2.63
16.	Lichen simplex chronicus	1	2.63
17.	Sore on cattle	1	2.63
18.	Dislocation of a body part	1	2.63
19.	Avian cholera	1	2.63
20.	Black leg	1	2.63
21.	Dysentery	1	2.63
22.	Diarrhea	1	2.63
23.	Bloating	1	2.63
24.	Piles	1	2.63
25.	Headache	1	2.63
26.	Kidney stone	1	2.63
27.	Hair loss	1	2.63
28.	Insects repellent	2	5.263
29.	Hairfall and thinning	1	2.63
30.	Alimentary tract and metabolic disorders, high blood pressure, common cold	1	2.63
31.	Cold, cough, and acute asthma	1	2.63
32.	Obesity	1	2.63
33.	Wound/skin cut	1	2.63
	Total	38	100%

The present study revealed that plant toxins, leech infections, scabies, and insect repellent use were the most common diseases treated in the area (Table 6). Plant toxins have been identified as a common concern in several regions, particularly in areas with rich biodiversity and traditional reliance on plants for medicinal purposes. In line with this, previous research by Smith [68] suggested that exposure to toxic plants and their inadvertent ingestion is a significant cause of illness in rural populations. The high incidence of plant toxin cases in our study is consistent with Johnson′s [69] findings, which documented frequent poisoning incidents due to the misuse or accidental consumption of native plants.

However, our study further emphasizes the importance of proper identification and knowledge regarding medicinal and toxic plants, which is often lacking in communities with limited access to healthcare or education. Leech infections, although seemingly a more specific condition, are also quite common in regions with abundant freshwater ecosystems (Table 6). Studies by Anderson [70] and Tan [71] found that leech infestations and subsequent infections are prevalent in humid, tropical climates. Our study concurs with these findings, noting that leech bites lead to infections, especially when proper wound care is not available. This highlights a need for improved public health initiatives focused on preventing leech bites, especially during the wet season when leech activity increases.

Scabies remains a widespread problem, particularly in areas with overcrowded living conditions or limited access to hygiene products (Table 6). In this context, the findings of Liu [72], which identified scabies as one of the most common dermatological conditions in rural areas, are in agreement with our results. The high treatment rates for scabies in our study reflect a broader global concern about this condition, as emphasized by Kaur and Sharma [73], who observed that scabies outbreaks are common in low‐resource settings where personal hygiene and sanitation are challenges. The frequent use of insect repellents noted in this study underscores the ongoing battle against vector‐borne diseases, particularly malaria, dengue, and other insect‐transmitted diseases (Table 6). Insect pests and parasitic organisms are major challenges that can cause diseases and significantly reduce livestock productivity. To minimize these problems, local communities and livestock producers traditionally use medicinal plants as natural insect repellents and treatments. These ethnoveterinary practices play an important role in protecting animal health, reducing production losses, and supporting sustainable livestock management. This corresponds with findings from Nguyen [74], who observed that the use of insect repellents and other preventive measures is critical in reducing the transmission of mosquito‐borne diseases in tropical regions. Our results corroborate this, highlighting the need for public health campaigns to raise awareness about the proper and consistent use of insect repellents as part of an integrated approach to vector control.

### 3.9. Mode of Application

The result indicated that oral route administration (51.28%) was the highest in number, followed by dermal route administration (30.77%) in the study area (Figure 5). The findings of this study reveal that the oral route was the most common mode of entry, followed by dermal, nasal, and spray methods (Figure 5). This result aligns with several previous studies on the commonality of different routes of drug administration or exposure to substances.

**Figure 5:**
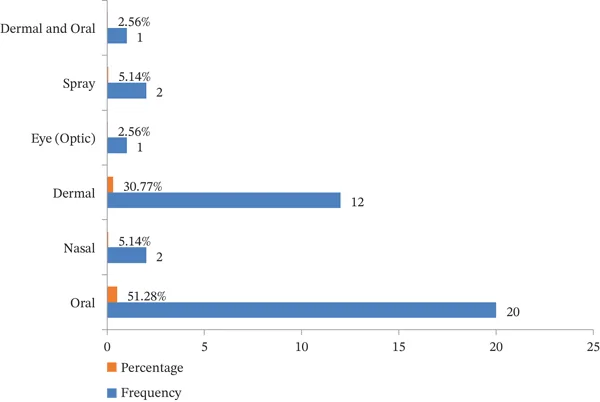
Mode of application.

The oral route, being the most prevalent mode of entry, is consistent with the vast body of literature supporting its widespread use due to its convenience, ease of administration, and patient preference. According to Smith [75], oral medications are the most commonly prescribed due to their affordability and the ability to administer them without requiring professional assistance. Additionally, the gastrointestinal absorption of many substances is well understood, making the oral route particularly reliable for controlled drug delivery. The dermal route, as the second most common, has seen increasing attention in recent studies, especially in the context of topical therapies and transdermal drug delivery systems. A study by Lee and Zhang [76] emphasized the importance of the skin as a barrier, yet it remains an effective route for systemic drug delivery due to the development of technologies that enhance permeation.

The dermal route also facilitates local treatment of conditions like skin diseases, making it highly relevant in certain therapeutic applications (Figure 5). The nasal route, although not as commonly used as oral or dermal, has gained recognition, especially in the delivery of vaccines and rapid‐onset drugs (Figure 5). As highlighted by Patel [77], the nasal route provides a noninvasive method for drug administration with a quicker onset due to the rich vascularization of the nasal mucosa. Despite its growing use in specific cases, the nasal route has limitations in terms of irritability and ease of administration, which might explain its lower frequency in our study. The spray method, though a popular delivery form in certain applications (e.g., nasal sprays or inhalers), had the lowest mode of entry in this study. Reviews by Karger [78] found that sprays are most often used in respiratory treatments or localized therapies but are less common overall when compared with other routes. The lower preference for spray methods might be due to the higher variability in dosage delivery and the need for proper techniques.

### 3.10. Most Important Medicinal Plant Species

#### 3.10.1. Informant Consensus

An informant consensus result helps to evaluate the proportion of informants who can maintain the use. The consequences of this study revealed that some medicinal plants were more familiar than others, and the highest informant consensus went to *E*. *cymosa* Thonn. and *Rhamnus prinoides* L′Herit., which were cited by informants as some use citation and taxa revealed in the study area (Table 7).

**Table 7:** Informants′ consensus factor (ICF) for more prevalent health ailments in the study area.

Categories of diseases	No. of use citation	Number of taxa	ICF
Increase milk production	90	50	0.5
Plant toxins	70	65	0.07
Leech infection	91	45	0.51
Insects repellent	79	35	0.56
Scabies	70	40	0.43

The familiarity of these medicinal plants was due to people′s preference for the species to treat different diseases in livestock. Ailments reported in the study area were grouped into different categories based on the site of occurrence of the disease, the condition of the disease, and treatment features in common. The informant′s consensus factor was calculated for each category. In the study, the informant consensus of medicinal plant usage resulted in ICF ranging from 0.56 to 0.43 per disease category for livestock health ailments (Table 7).

#### 3.10.2. Preferences for Livestock Medicinal Plants

The given medicinal plant species were used in the treatment of divergent illnesses, but local people showed a preference towards a plant species with a healing potential for various ailments. Preference ranking of eight selected medicinal plant species on the basis of treating several ailments showed *Cymbopogon citratus* (DC.) Stapf as the most preferred one, followed by *Euphorbia cactus* Ehrenb. ex Boiss. and *Clitoria ternatea* L. as the least preferred one. Larger value (8) was analyzed for species having great healing potential, (7) for species considered to have the next healing power, and the smallest value (1) for species having low healing potential as compared with other species (Table 8).

**Table 8:** Preference ranking of eight medicinal plants for any medicinal value.

Plant species name	Ranking value for each respondent (A–G)							Total	Rank
	A	B	C	D	E	F	G		
*Clitoria ternatea* L.	3	1	2	1	2	1	2	12	8th
*Bryophyllum pinnatum* (Lam.) Oken.	4	6	5	6	5	5	5	36	4th
*Datura stramonium* L.	2	2	1	2	1	2	3	13	7th
*Solanum trilobatum* L.	5	5	3	3	4	3	4	27	5th
*Euphorbia cactus* Ehrenb. ex Boiss.	6	4	8	8	8	8	6	48	2nd
*Rhamnus prinoides* L′Herit.	1	3	4	4	3	4	1	20	6th
*Croton macrostachyus* Hochst. ex Delile.	7	7	6	5	6	6	8	45	3rd
*Cymbopogon citratus* (DC.) Stapf.	8	8	7	8	7	7	7	52	1st

#### 3.10.3. DMR

##### 3.10.3.1. Ranks of Multipurpose Medicinal Plants

The result of DMR revealed that *Eucalyptus globulus* Labill., *Phytolacca dodecandra* L′Herit., *Croton macrostachyus* Hochst. ex Delile., *Dodonaea angustifolia* L. f., *C*. *citratus* (DC.) Stapf, *Cissus quadrangularis* L., *Calpurnia aurea* (Alt.) Benth., and *Solanum marginatum* L. f. were ranked 1st–8th, respectively (Table 9). Pooling of the direct ranking matrix on five use categories revealed that medicine, furniture, charcoal, tools, and building were ranked first, second, third, fourth, and fifth, respectively (Table 9).

**Table 9:** Direct matrix ranking of eight plant species based on five use criteria.

Species	Use criteria					Total	Rank
	C	MD	BU	FU	TO		
*Solanum marginatum* L. f.	1	7	1	1	1	12	8th
*Cissus quadrangularis* L.	2	2	2	8	1	15	6th
*Calpurnia aurea* (Alt.) Benth.	2	2	2	4	3	13	7th
*Croton macrostachyus* Hochst. ex Delile.	5	4	6	7	5	27	3rd
*Phytolacca dodecandra* L′Herit.	4	7	7	7	4	29	2nd
*Cymbopogon citratus* (DC.) Stapf	7	3	2	3	7	22	5th
*Dodonaea angustifolia* L. f.	7	6	4	3	6	26	4th
*Eucalyptus globulus* Labill.	9	8	8	6	8	39	1st
Total	37	39	32	38	35		
Rank	3rd	1st	5th	2nd	4th		

Results of informants against eight use values other than their food use: C = charcoal, MD = medicines, BU = building, FU = furniture, and TO = tools.

#### 3.10.4. FL

##### 3.10.4.1. Relative Healing Potential of Medicinal Plants for Livestock Ailments

The result showed *P*. *dodecandra* L′Herit., *R*. *prinoides* L′Herit., *Ruta chalepensis* L., *Allium sativum* L., and *N*. *tabacum* L. were an indication of their good healing potential in the study area, followed by *S*. *marginatum* L. f. and *Ricinus communis* L. (Table 10).

**Table 10:** Fidelity level value indexes of traditional medicinal plants to treat disease.

Plant species	Treated ailment	IP	IU	FL value	FL (%)
*Phytolacca dodecandra* L′Herit.	Rabies	6	5	1	100%
*Rhamnus prinoides* L′Herit.	Toxicosis	6	6	1	100%
*Nicotiana tabacum* L.	Leech infestation	3	4	0.75	75%
*Ruta chalepensis* L.	Coccsidiosis	5	5	1	100%
*Solanum marginatum* L. f.	Urinary retention	2	3	0.66	66%
*Ricinus communis* L.	Anthrax	1	2	0.5	50%
*Allium sativum* L.	Thelaziasis	9	10	0.9	90%

## 4. Conclusion

The results of this study are generally in line with previous research on plant family distribution, confirming the prominence of Solanaceae, Euphorbiaceae, and Fabaceae in disturbed or semidisturbed environments. These families′ ecological roles, such as resilience to disturbances and soil improvement through nitrogen fixation, highlight their importance in maintaining ecosystem function. Further studies are needed to explore the mechanisms driving the distribution of these families across different environmental conditions. The predominance of leaves in this study is consistent with existing literature, confirming their widespread utilization across various domains, particularly in traditional medicine and food. Although fruits, bulbs, and whole plants are also important, their lower representation might reflect specific cultural preferences or the practical challenges of utilizing these parts. Further studies could explore regional variations in plant part use to gain a deeper understanding of plant species′ roles in different societies.

The plant habit forms observed in this study reflect ecological processes such as succession and disturbance dynamics. Although herbs are more prominent in disturbed ecosystems or early successional stages, shrubs and trees are often more abundant in mature, undisturbed environments. The mode of entry hierarchy identified in this study, oral, dermal, nasal, and spray, reflects a global trend in drug delivery methods. Although oral administration remains the most commonly used and preferred route, dermal and nasal routes are becoming increasingly relevant, particularly with advancements in drug formulation technologies.

This study′s findings on plant toxins, leech infections, scabies, and insect repellent use are consistent with prior research, reinforcing the importance of these health concerns in specific populations. The results emphasize the need for targeted healthcare strategies, education, and preventive measures to address these prevalent issues effectively. The results of this study are generally in line with previous research on plant family distribution, confirming the prominence of Solanaceae, Euphorbiaceae, and Fabaceae in disturbed or semidisturbed environments. These families′ ecological roles, such as resilience to disturbances and soil improvement through nitrogen fixation, highlight their importance in maintaining ecosystem function. Further studies are needed to explore the mechanisms driving the distribution of these families across different environmental conditions.

### 4.1. Recommendations

Based on the findings and observations of this study on the ethnobotanical use of medicinal plants for the treatment of livestock ailments, the recommendations are as follows:

✓ Future studies should enable comparative analyses of ethno‐veterinary knowledge across different communities. This can help identify regional similarities and unique practices in traditional livestock treatment.
✓ Ethnobotanical research should integrate pharmacology, ecology, and anthropology to validate the efficacy and safety of medicinal plants.
✓ Traditional knowledge provides valuable insight; there is a critical need to scientifically validate the bioactive compounds of the most commonly used medicinal plants. This includes phytochemical screening, antimicrobial tests, and toxicity studies.
✓ Researchers should prioritize documentation of traditional knowledge in detail, using participatory methods that engage local healers and livestock owners.
✓ Future research should assess the sustainability of the current harvesting methods and promote the cultivation of key species to ensure long‐term availability.
✓ Ethical guidelines should be followed, especially regarding intellectual property rights and the protection of indigenous knowledge
✓ Youth researchers should develop educational and outreach programs that encourage young participation and intergenerational knowledge transfer within the communities.
✓ By highlighting the efficacy and accessibility of traditional remedies, ethnoveterinary medicine can be integrated into primary animal healthcare systems, particularly in rural and underserved areas.

### 4.2. Limitation of the Study

The study did not collect detailed information on whether the treated animals were adults or young, nor did it document the specific dosages and quantities of medicinal plants administered. Consequently, the findings cannot assess age‐specific treatment practices or dosage standardization. Future ethnoveterinary studies should include these variables to improve the accuracy, reproducibility, and practical application of traditional animal healthcare knowledge. Beside these, the present study documented indigenous ethnoveterinary knowledge but did not evaluate the pharmacological efficacy, safety, or toxicity of the reported medicinal plants. Therefore, further ethnopharmacological studies are needed to scientifically validate the therapeutic potential and appropriate dosages of these traditional remedies.

## Author Contributions

**Tamirat Wato:** conceptualization, investigation, methodology, software, formal analysis, data curation, writing – original draft. **Nigus Kalayou:** conceptualization, investigation, methodology, software, formal analysis, data curation, writing – original draft. **Abera Haile:** resources, investigation, data curation, writing – review & editing. **Salasib Atumo:** resources, investigation, data curation, writing – review & editing. **Alemu Andualem:** resources, investigation, data curation, writing – review & editing. **Ginjo Gitima:** resources, investigation, data curation, writing – review & editing. **Mathewos Agize:** methodology, writing – review & editing, validation, data curation.

## Funding

No funding was received for this manuscript.

## Conflicts of Interest

The authors declare no conflicts of interest.

## Data Availability

The data that support the findings of this study are available on request from the corresponding author. The data are not publicly available due to privacy or ethical restrictions.
